# A Cerebrovascular Accident in the Setting of Kingella kingae Infective Endocarditis: A Case Report

**DOI:** 10.7759/cureus.88668

**Published:** 2025-07-24

**Authors:** Aneil S Walizada, Arianna Lozada, Dania Rizwan, Nathan Zaher

**Affiliations:** 1 Internal Medicine, Hospital Corporation of America (HCA) Florida Westside Hospital, Plantation, USA

**Keywords:** cerebrovascular accident, hacek, infective endocarditis, kingella kingae, septic emboli

## Abstract

*Kingella kingae*, a rare member of the *Haemophilus*, *Aggregatibacter*, *Cardiobacterium*, *Eikenella*, and *Kingella* (HACEK) group, is an uncommon cause of infective endocarditis (IE) in adults. Advances in molecular diagnostics, including polymerase chain reaction (PCR) and matrix-assisted laser desorption/ionization time-of-flight mass spectrometry (MALDI-TOF MS), have significantly improved its identification. We present the case of a 78-year-old immunocompromised woman who developed watershed territory cerebral infarctions in the setting of *K. kingae* endocarditis. The patient initially presented with fever, altered mental status, and right-sided weakness and was later found to have *K. kingae *bacteremia, as well as a 7 mm aortic valve vegetation. Due to high surgical risk, the patient was managed conservatively with long-term intravenous antibiotics. This case highlights how modern diagnostic tools facilitate early detection of atypical pathogens and allow for more timely non-surgical management in vulnerable populations.

## Introduction

*Kingella kingae*, a gram-negative coccobacillus of the *Haemophilus*, *Aggregatibacter*, *Cardiobacterium*, *Eikenella*, and *Kingella* (HACEK) group, is commonly associated with osteoarticular infections in children but remains a rare cause of infective endocarditis in adults [1-3]. The reported incidence of HACEK-related IE is low, constituting approximately 1-3% of all IE cases [4]. *K. kingae* IE is especially uncommon in older adults and often underrecognized due to diagnostic challenges and due to its slow growth in standard cultures [3,5]. Advances such as prolonged incubation protocols, PCR assays, and MALDI-TOF MS, however, have significantly enhanced detection of this organism, enabling earlier diagnosis and treatment [5-7].

This case report discusses *K. kingae* as a possible pathogen in immunocompromised adults presenting with sepsis and neurologic symptoms, emphasizing the role of modern microbiologic techniques in timely diagnosis.

## Case presentation

A 78-year-old woman with a history of cholangiocarcinoma undergoing chemotherapy and immunotherapy (nivolumab), deep vein thrombosis, atrial fibrillation on apixaban, prior cerebrovascular accident without residual deficits, chronic kidney disease stage 3b, hypertension, type 2 diabetes mellitus, and heart failure with preserved ejection fraction presented with fever, altered mental status, and new-onset right-sided weakness. Her last chemotherapy cycle occurred 10 days prior to arrival.

On presentation, vital signs were significant for a temperature of 38.6 degrees Celsius (101.4 degrees Fahrenheit), a pulse of 122 beats per minute, and a blood pressure of 182/76 mmHg. The patient met systemic inflammatory response syndrome (SIRS) criteria and was treated empirically with ceftriaxone and fluids. Initial labs showed no leukocytosis or lactic acidosis. Blood cultures were collected for ongoing monitoring.

**Figure 1:**
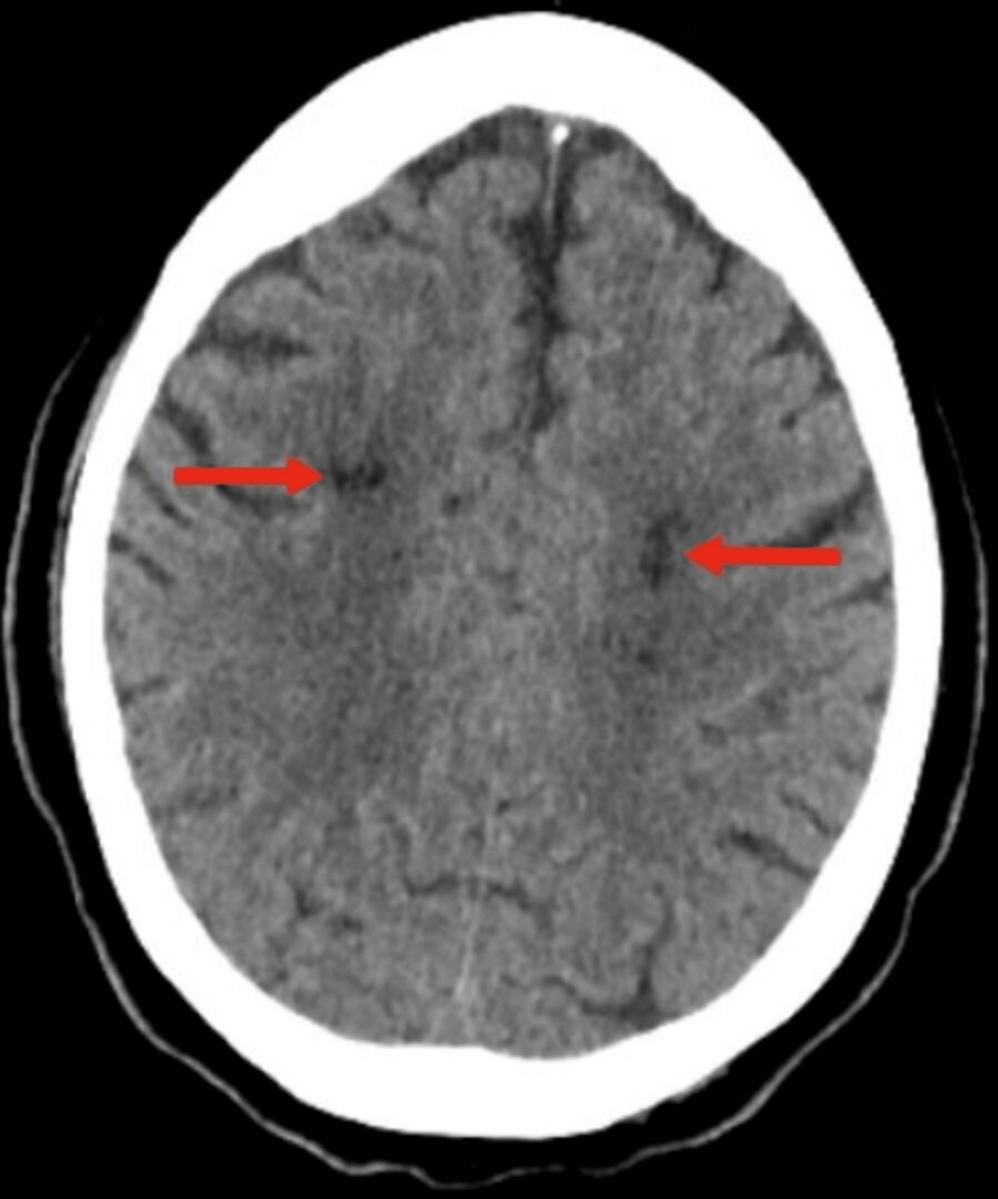
Computerized tomography (CT) scan of the brain without contrast revealing sequelae of prior infarcts (red arrows).

A stroke alert was initiated due to focal neurological deficits. The patient was ineligible for tissue-type plasminogen activator (tPA) due to receiving apixaban at home. Head CT showed no hemorrhage but evidence of prior infarcts (Figure 1).

**Figure 2:**
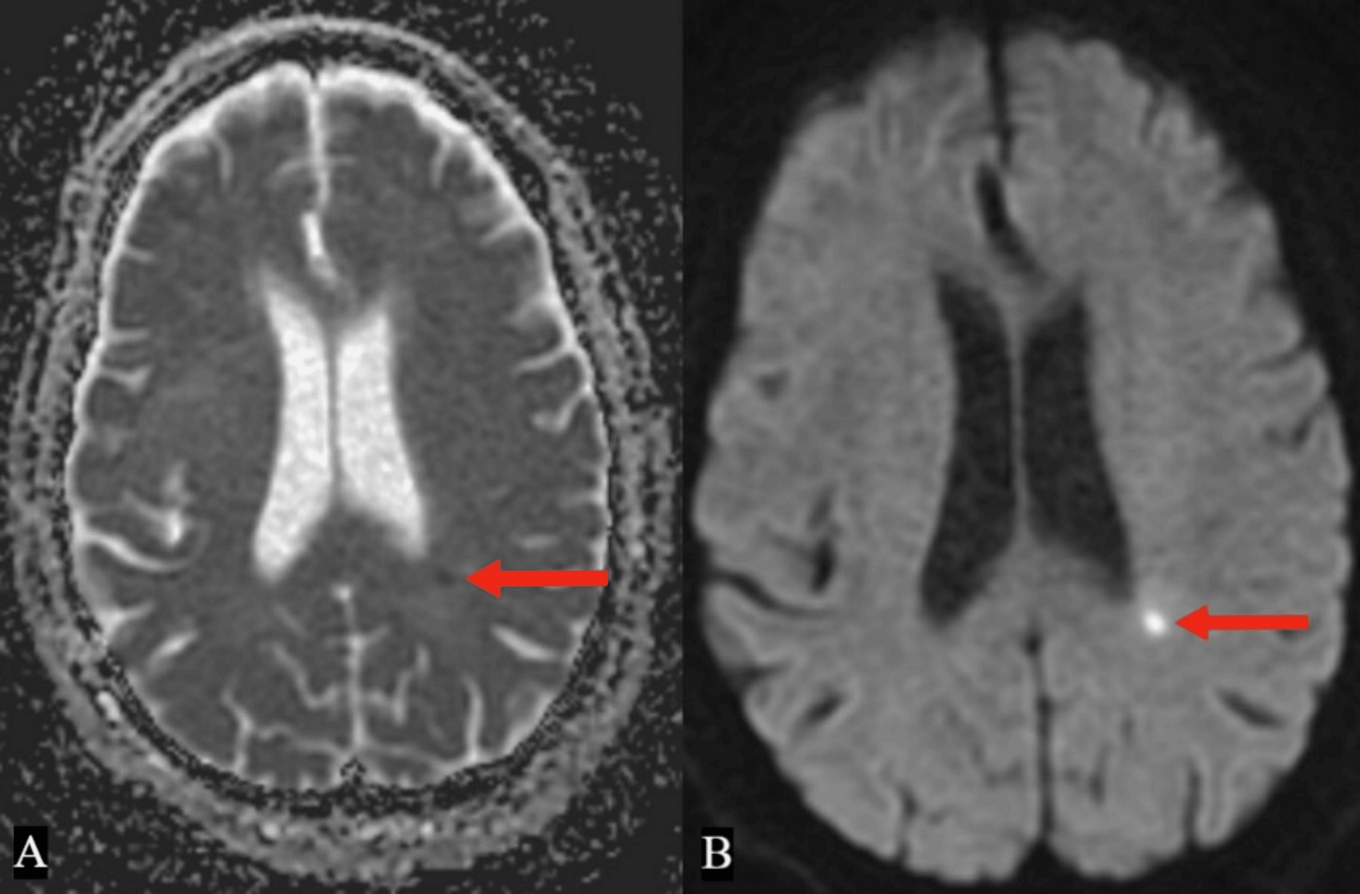
Magnetic resonance imaging (MRI) of the brain revealing subacute watershed territory infarctions (red arrows). Figure 2A: MRI brain with apparent diffusion coefficient mapping. Figure 2B: MRI brain with diffusion-weighted imaging.

Brain MRI demonstrated subacute infarcts in bilateral watershed territories without midline shift or hemorrhage (Figure 2).

These infarcts are typical of hypoperfusion, but the differential also included cardioembolism in the setting of atrial fibrillation, carotid stenosis, and echocardiographic findings.

**Figure 3:**
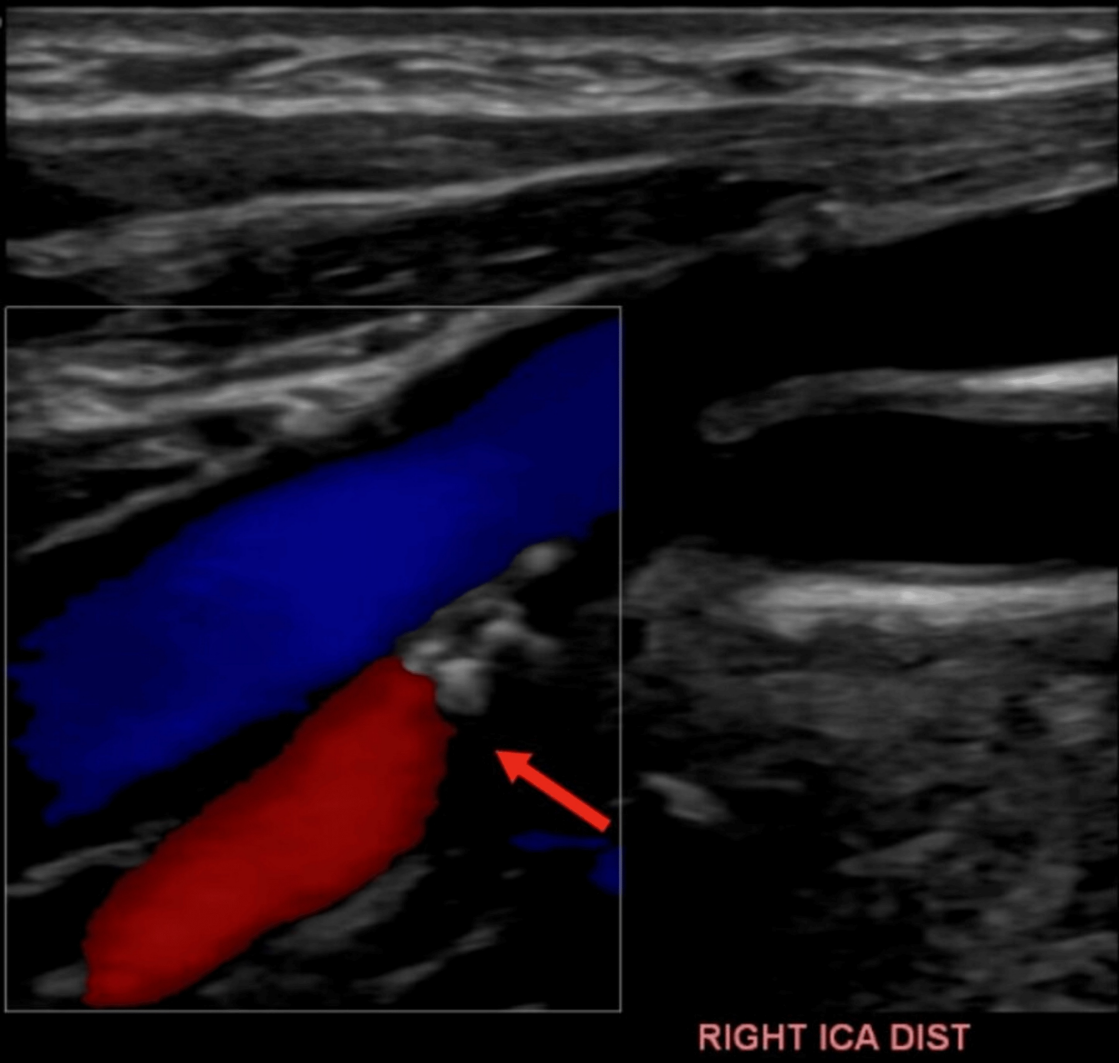
Ultrasound doppler of the bilateral carotid arteries revealed 50-69% stenosis of the right internal carotid artery (red arrow).

Carotid duplex revealed 50-69% stenosis of the right internal carotid artery (Figure 3).

A CT angiogram of the head and neck ruled out large vessel occlusion or aneurysms, while a CT chest ruled out pulmonary sources of emboli.

**Figure 4:**
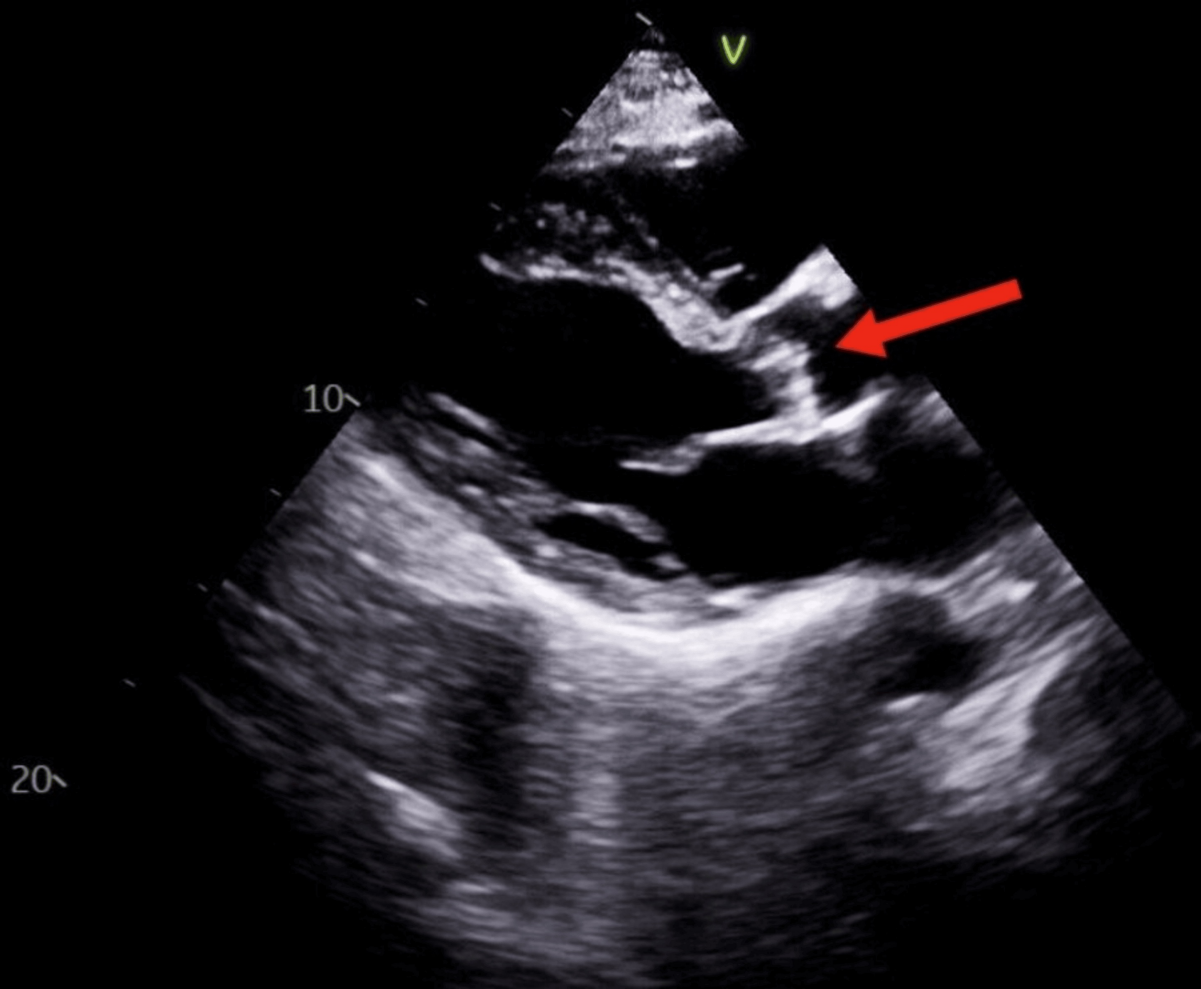
Transthoracic echocardiogram (TTE) in the parasternal long axis view showing a 7 mm vegetation of the aortic valve (red arrow).

A transthoracic echocardiogram (TTE) showed dilated atria and moderate regurgitation across the aortic and tricuspid valves, with a suspicious 7 mm mobile vegetation on the aortic valve (Figure 4).

Transesophageal echocardiography confirmed the aortic valve vegetation, without evidence of abscess or perforation.

Two sets of blood cultures grew *Kingella kingae*, identified via MALDI-TOF MS. Repeat cultures remained negative after antibiotic initiation. Given the organism’s clinical relevance, repeated isolation, compatible echo, and clinical findings, and the patient's immunocompromised state, contamination was considered unlikely.

The patient initially received vancomycin but was transitioned to intravenous ceftriaxone 2 g daily for six weeks. Surgery was deferred due to high perioperative risk.

## Discussion

Infective endocarditis in older adults is typically caused by *Staphylococcus aureus* or *Streptococcus* species; however, *K. kingae*, a HACEK organism often associated with pediatric osteoarticular infections, has also been found as a cause, particularly in immunocompromised hosts [2,3]. Management of IE depends on patient health, valve type, and complications such as heart failure or embolic events [4]. Strokes secondary to hypoperfusion often present as watershed infarctions on MRI; however, embolic causes of stroke can at times present similarly [8]. Early recognition of neurological symptoms is essential for guiding treatment decisions.

Empiric antibiotic therapy remains the cornerstone of initial management in suspected infective endocarditis, ensuring timely coverage until blood cultures identify the causative organism [4,7]. In cases of persistent bacteremia, valve perforation, or recurrent embolic events, however, surgical intervention is generally considered despite antibiotic therapy [9,10]. Treatment decisions depend on surgical risk and ongoing embolization, with conservative management preferred in high-risk patients [9-11].

This case emphasizes the importance of considering *K. kingae* in culture-negative infective endocarditis, especially in immunocompromised patients presenting with sepsis and stroke-like symptoms. Modern diagnostic techniques have significantly improved the early detection and treatment of HACEK-related infective endocarditis, which has enhanced the likelihood of successful medical management and reduced the risk of complications such as septic emboli and stroke [5-7]. 

Neurologic complications of IE are significant. Mobile vegetations >10 mm are associated with higher embolic risk [4,11]. The patient's 7 mm mobile vegetation likely contributed to embolism, though watershed infarcts also suggest hypoperfusion as an etiology. Atrial fibrillation and carotid stenosis remain alternative stroke sources. Neurologic complications of infective endocarditis may occur even in the absence of emboli on vascular imaging, underscoring the diagnostic complexity in these patients [12,13].

Surgical intervention is often considered for IE complicated by embolic stroke, but in this case, the patient’s malignancy, immunocompromised state, and comorbidities made her a poor surgical candidate. As such, long-term therapy with intravenous ceftriaxone was pursued, consistent with recommendations for medically managing HACEK IE when surgery is contraindicated [9-11].

## Conclusions

Early identification of *Kingella kingae* using prolonged culture incubation, MALDI-TOF MS, and PCR is vital for timely, pathogen-specific therapy. Although rare in adults, *K. kingae* can cause severe complications, including stroke, particularly in immunocompromised patients. A broad differential and awareness of HACEK organisms are important in culture-negative endocarditis. Nonsurgical management with appropriate antibiotics can yield favorable outcomes when surgery is not feasible in high-risk patients.
